# Clinical Outcomes of Endoscopic Submucosal Dissection for Early Gastric Cancer Patients With Concurrent Advanced Malignancies With Expected Poor Prognosis

**DOI:** 10.1002/jgh3.70226

**Published:** 2025-07-15

**Authors:** Hiroki Takemoto, Hidehiko Takigawa, Takahiro Kotachi, Hajime Teshima, Akiyoshi Tsuboi, Hidenori Tanaka, Ken Yamashita, Yuji Urabe, Toshio Kuwai, Shiro Oka

**Affiliations:** ^1^ Department of Gastroenterology Hiroshima University Hospital Hiroshima Japan; ^2^ Gastrointestinal Endoscopy and Medicine Hiroshima University Hospital Hiroshima Japan

**Keywords:** advanced malignant tumor, early gastric cancer, endoscopic submucosal dissection, multiple primary cancer

## Abstract

**Aims:**

Despite improved outcomes for malignant tumors, evidence regarding the management of patients with multiple malignancies remains limited. We aimed to evaluate the clinical outcomes and prognosis of patients undergoing endoscopic submucosal dissection (ESD) for early gastric cancer (EGC) complicated by advanced malignancies in other organs with a poor prognosis.

**Methods and Results:**

We retrospectively reviewed 3703 gastric cancer patients who underwent ESD at our hospital (2005–2022), focusing on those with advanced extra‐gastric malignancies with a 5‐year survival rate of < 50%. ESD was performed for local tumor control based on patient preference when feasible, including lesions meeting standard, expanded, or relative indications where curative resection was unachievable. Clinicopathological characteristics and outcomes were analyzed. Twenty‐six patients met the inclusion criteria. En bloc resection was achieved in all cases (100%), with curative and non‐curative resection in 16 (62%) and 10 (38%) cases, respectively. None of the 10 patients with non‐curative resection exhibited lymphovascular invasion or GC recurrence. Complications included delayed bleeding, perforation, and pneumonia, each in one patient (4%), all leading to disseminated intravascular coagulation (DIC) and death within 30 days post‐ESD. Notably, no complications were reported after 2010. Eleven patients died from advanced malignant tumors, with no GC recurrences observed during follow‐up in surviving patients.

**Conclusions:**

Recently, no severe complications have been observed with ESD. Although ESD for local control in EGC with concurrent advanced extra‐gastric malignancies may be acceptable, the risk of severe complications, including DIC, remains. Therefore, careful patient selection and thorough informed consent are essential.

## 1 Introduction

Recently, notable progress has been made in malignant tumor treatment, including advances in minimally invasive surgery [1], the development of chemotherapy options such as immune checkpoint inhibitors [2], and the introduction of drugs to mitigate chemotherapy‐induced side effects [3]. Consequently, patient prognosis has improved, leading to an increase in the number of individuals diagnosed with multiple primary malignant tumors. However, there is currently no evidence‐based guidance on the optimal treatment strategies for patients with multiple primary malignant tumors.

Gastric cancer (GC) remains highly prevalent in East Asian countries, including Japan, Korea, and China [4, 5]. Early gastric cancer (EGC) complicated by advanced malignant tumors of other organs presents a clinical challenge, particularly in determining appropriate treatment indications. Factors such as patient performance status (PS) play a crucial role in treatment decisions, as those with low PS may not be suitable candidates for surgery. In such instances, the prognosis of the advanced malignancy often becomes a key factor in determining the course of treatment for coexisting EGC.

Endoscopic submucosal dissection (ESD) has become a widely accepted, minimally invasive therapy for EGC, offering organ preservation and improved quality of life, especially in older patients [6]. However, evidence regarding ESD efficacy and safety in patients with advanced malignancies of other organs remains limited. Previous studies, such as the one conducted by Akasaka et al., have shown ESD to be relatively safe, with low rates of perforation, postoperative bleeding, and pneumonia, and no reported deaths [7]. Nevertheless, EGC treatment in patients with advanced malignancies in other organs should be carefully considered, particularly when the prognosis of the advanced GC of other organs is expected to be better than that of EGC. The natural history of untreated EGC has shown that the median time for progression to advanced carcinoma is 34 [8] to 44 [9] months, with a 5‐year corrected survival rate of 62.8% for unresected cases [9]. In another study analyzing 12 cases, EGC progressed to advanced cancer within a median of 9.6 months, with a 5‐year survival rate of 45% [10].

Accordingly, ESD treatment for EGC in patients with advanced malignancies and a 5‐year survival rate exceeding 50% is considered a reasonable approach. However, in cases where the advanced malignancy has a poor prognosis, with a 5‐year survival rate below 50%, the malignancy itself may be the primary prognostic factor. In such cases, determining whether to treat the coexisting EGC can be challenging.

EGC that coexists with advanced malignancies in other organs with a 5‐year survival rate below 50% may not directly cause death, but instead can progress to advanced GC over time. This progression may lead to complications such as bleeding, obstruction, or anemia, potentially interfering with chemotherapy for the advanced malignancy [11]. Given the poor surgical tolerance in these patients, ESD may be a reasonable option to prevent such complications. However, EGC treatment may not always be appropriate depending on the overall prognosis. We retrospectively analyzed patients with EGC with primary malignancies in other organs (5‐year survival < 50%, PS 0–1). ESD was performed based on patient preference in cases where local endoscopic resection was deemed feasible. This included not only lesions meeting the absolute indications or expanded indications for ESD but also those with relative indications where curative resection was considered unachievable.

This study aimed to evaluate the clinical outcomes and prognosis of ESD in patients with EGC with advanced primary malignancies in other organs with a 5‐year survival rate of < 50% and to assess the risks and benefits of ESD in this complex patient population.

## 2 Methods

### 2.1 Study Design and Patients

**FIGURE 1:**
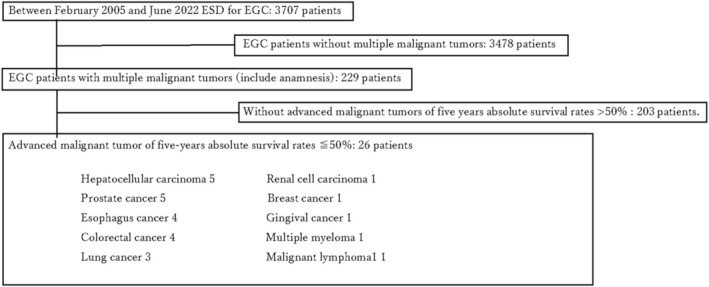
Flowchart of enrolled patients. From the 3707 patients who underwent ESD for EGC between February 2005 and January 2022 at our hospital, 3478 patients without multiple malignant tumors were excluded. The total number of patients with multiple malignant tumors was 229. Furthermore, patients with a 5‐year absolute survival rate of more than 50% were excluded. This resulted in 26 patients with a 5‐year absolute survival rate of < 50%. The following table provides further details: five patients with hepatocellular carcinoma, five with prostate cancer, four with esophageal cancer, four with colon cancer, three with lung cancer, one with renal cell cancer, one with breast cancer, one with supragingival cancer, one with multiple myeloma, and one with malignant lymphoma (diffuse large B‐cell lymphoma; DLBCL). ESD, endoscopic submucosal dissection; EGC, early gastric cancer.

In this retrospective study, we enrolled 26 patients with advanced malignant tumors of other organs, including metachronous cancer. All patients had a 5‐year absolute survival rate of < 50%. These patients were selected from a cohort of 3703 individuals with GC who underwent ESD at our hospital between February 2005 and June 2022 (Figure 1). Clinicopathological characteristics and ESD outcomes were evaluated. Definitions of multiple primary malignant tumors were based on criteria established by Warren and Gates in 1932: (1) histological confirmation of malignancy for each neoplasm; (2) distinct pathomorphological features for each neoplasm; (3) development of neoplasms in different locations without connections; and (4) exclusion of metastasis [12]. The 5‐year absolute survival rate was determined based on a Japanese report [13]; the TNM staging was classified according to guidelines of relevant Japanese societies [14, 15, 16, 17, 18, 19, 20].

The study included patients with EGC who were deemed eligible for endoscopic local resection. This included lesions meeting not only the absolute indications or expanded indications for ESD but also relative indications where curative resection was considered unachievable.

Patients were thoroughly informed about the risks associated with ESD before performing the procedure. In particular, for lesions meeting the criteria for relative indications, patients were explicitly informed about the possibility of non‐curative resection and the significance of local disease control. ESD was performed only for those who fully understood these considerations and opted for the procedure.

### 2.2 Specimen Assessment

Gastric lesions were categorized into three sections: upper third (U), middle third (M), and lower third (L) of the stomach [21]. Macroscopic types were classified as protruding (0–I), superficial (0–II), or excavated (0–III), with the superficial type further divided into superficially elevated (0–IIa), superficially flat (0–IIb), and superficially depressed (0–IIc). For tumors exhibiting ≥ 2 macroscopic types, the classification was based on the type occupying the largest surface area (e.g., 0–IIa + IIc) [21]. Patient characteristics (age, sex, advanced malignancy, and clinical stage), endoscopic findings, pathological features (tumor size, location, macroscopic type, histology, tumor invasion depth, ulceration, lymphovascular invasion, and resection margins), and ESD outcomes (en bloc resection, curative resection, noncurative resection, procedure time, adverse events, and outcomes) were documented.

### 2.3 Histopathological Evaluation

Following resection, a histopathological examination was performed according to the Japanese Classification of Gastric Carcinoma [22]. Histological types were categorized into differentiated‐type adenocarcinoma (tubular and papillary adenocarcinoma) and undifferentiated‐type adenocarcinoma (poorly differentiated adenocarcinoma, signet‐ring cell carcinoma, and mucinous adenocarcinoma) [23]. Submucosal tumor invasion (T1b) was subclassified as SM1 (< 500‐μm invasion from the muscularis mucosa) or SM2 (≥ 500‐μm invasion) [23]. The assessment included ulcer examination, scar findings, and lymphovascular invasion.

### 2.4 Pathological Curability

The entire resection for an en bloc‐resected tumor is regarded as all vertical and lateral margins with no tumors during histological examination [22].

A lesion is considered endoscopic curability A (eCuraA) when it is resected en bloc and meets the following conditions [21]: (i) predominantly differentiated type, pT1a, UL0, HM0, VM0, Ly0, V0, regardless of size; (ii) long diameter ≤ 2 cm, predominantly undifferentiated type, pT1a, UL0, HM0, VM0, Ly0, V0; or (iii) long diameter ≤ 3 cm, predominantly differentiated type, pT1a, UL1, HM0, VM0, Ly0, and V0.

A lesion is considered endoscopic curability B (eCuraB) when it is resected en bloc, has a long diameter ≤ 3 cm, is predominantly of the differentiated type, and satisfies the following criteria: pT1b1 (SM1) (within < 500 μm from the muscularis mucosae), HM0, VM0, Ly0, and V0.

When a lesion meets neither eCuraA nor B conditions, it is considered eCuraC, corresponding to the concept of non‐curative resection.

### 2.5 Assessment of Complications

This study evaluated perforations during ESD, delayed bleeding, delayed perforation, and postoperative pneumonia. Delayed bleeding was defined as any of the following: an episode of hematemesis and/or melena, a drop of 2 g/dL in hemoglobin level, and a suspicion of bleeding requiring a new endoscopy within 30 days after ESD completion. Delayed perforation was defined as cases wherein perforation had not been detected during and just after ESD completion, but subsequent endoscopy showed perforation or computed tomography (CT) showed free air post‐ESD. Postoperative pneumonia was defined as fever, decreased oxygen saturation, or pneumonia on imaging.

### 2.6 Clinical Outcome Assessment

Clinical outcomes were retrospectively collected from medical records from February 2005 to June 2022. Causes of death were classified as related to advanced malignant tumors of other organs, other causes, or ESD‐related. ESD‐related death was defined as death occurring within 30 days post‐ESD.

## 3 Results

### 3.1 Patient Characteristics

Between February 2005 to June 2022, 3703 patients with EGC underwent ESD at our hospital. Among these, 229 patients had multiple malignant tumors, including metachronous malignant tumors; of these, 26 patients had advanced malignant tumors in other organs with a 5‐year absolute survival rate of < 50% (Figure 1). Characteristics of these 26 patients are summarized in Table 1. The median age was 75 years (range 50–90 years); 81% (21/26) of patients were male. The distribution of advanced cancers included five cases of hepatocellular carcinoma (stage II in 4, stage IV in 1), five of prostate cancer (all stage IV), four of esophageal cancer (stage II in 2, stage IV in 2), four of colon cancer (all stage IV), three of lung cancer (all stage IV), and one each of renal cell carcinoma (stage IV), breast cancer (stage IV), supragingival cancer (stage IV), multiple myeloma (stage III), and malignant lymphoma (diffuse large B‐cell lymphoma, stage III). The clinical stages of these cancers were stage II in 6 (23%), stage III in 2 (8%), and stage IV in 18 (69%) patients. All 26 patients were undergoing treatment or pre‐treatment and had good PS, with 69% (18/26) having PS 0 and the remaining (8/26) having PS 1.

**TABLE 1:** Patient characteristics.

Characteristic, *n* (%)	(*n* = 26)
Median age, years [range]	75.0 [50–90]
Sex, male	21 (81)
Advanced malignant tumor	
Hepatocellular carcinoma	5 (19)
Prostate cancer	5 (19)
Esophagus cancer	4 (15)
Colorectal cancer	4 (15)
Lung cancer	3 (12)
Renal cell carcinoma	1 (4)
Breast cancer	1 (4)
Gingival cancer	1 (4)
Multiple myeloma	1 (4)
Malignant lymphoma	1 (4)
cStage	
cStage II	5 (19)
cStage III	2 (8)
cStage IV	19 (73)
ECOG performance status (PS)	
PS 0	18 (69)
PS 1	8 (31)

Abbreviations: cStage, clinical stage; ECOG, Eastern Cooperative Oncology Group.

### 3.2 Endoscopic Findings and Pathological Characteristics

Endoscopic and pathological characteristics are detailed in Table 2. The median tumor size was 20 mm (range 5–110 mm). The tumor location was distributed as follows: U in six lesions (23%), M in six lesions (23%), and L in 14 lesions (54%). Macroscopically, two lesions (7%) were of the protruding type, 10 (35%) were superficial elevated type, and 14 (58%) were superficial depressed type. Histologically, 88% (23/26) of tumors were of the differentiated type, whereas 12% (3/26) were of the undifferentiated type. Invasion depth was pT1a in 18 (69%), pT1b1 in three (12%), and pT1b2 in five (19%) cases. Ulcerative findings were observed in one lesion (4%), and one lesion had a positive vertical margin (VM). All lesions tested negative for lymphovascular invasion and horizontal margins.

**TABLE 2:** Endoscopic findings and pathological characteristics.

Variable, *n* (%)	(*n* = 26)
Median size, mm [range]	20.0 [5–110]
Location	
U	6 (23)
M	6 (23)
L	14 (54)
Macroscopic type	
Protruding type (0–I)	2 (7)
Superficial elevated type (0–II)	10 (35)
Superficial depressed type (0–IIc)	14 (48)
Major history	
Differentiated type	23 (89)
Undifferentiated type	3 (11)
Depth of tumor	
pT1a	18 (69)
pT1b1	3 (12)
pT1b2	5 (19)
Ulcerative findings	1 (4)
Lymphovascular invasion	0 (0)
Horizontal margin	0 (0)
Vertical margin	1 (4)

Abbreviations: L, lower; M, middle; pT1a, pathological T1a; pT1b1, pathological T1b1; pT1b2, pathological T1b2; U, upper.

### 3.3 ESD Outcomes

Table 3 outlines ESD outcomes. En bloc resection was successfully performed for all lesions. Curative resection was achieved in 16 lesions (62%), whereas 10 lesions (38%) required non‐curative resection. The non‐curative resection included cases with pT1b2 (*n* = 5), ulcerative findings with tumor size > 30 mm (*n* = 1), undifferentiated type with tumor size > 20 mm (*n* = 3), and positive VM (*n* = 1). The median procedure time was 66 min (range 10–330 min). Adverse events occurred in three cases (12%): delayed bleeding (*n* = 1), delayed perforation (*n* = 1), and postoperative pneumonia (*n* = 1). Clinical outcomes included death from advanced cancer in 11 patients (42%), death from ESD‐related adverse events in three patients (12%), death from other diseases in three patients (12%), and survival in nine patients (34%). No deaths were directly attributable to EGC.

**TABLE 3:** Outcomes of endoscopic submucosal dissection for early gastric cancer.

Variable, *n* (%)	(*n* = 26)
En block resection	26 (100)
Curative resection	16 (62)
Non‐curative resection	10 (38)
pT1b2	5 (19)
UL1, > 30 mm	1 (4)
Undifferentiated type, > 20 mm	3 (11)
VM1	1 (4)
Median, procedure time, min [range]	66 [10–300]
Adverse events	
Perforation during ESD	0 (0)
Delayed bleeding	1 (4)
Delayed perforation	1 (4)
Postoperative pneumonia	1 (4)
Outcomes	
Death due to original malignant tumor	11 (42)
Death due to other causes	3 (12)
ESD‐related death	3 (12)
Survival	9 (34)

Abbreviations: ESD, endoscopic submucosal dissection; pT1b2, pathological T1b2; UL, ulcerative findings; VM, vertical margin.

Table 4 details three patients who died from complications. Notably, these events occurred only in procedures before 2010; in 23 procedures after 2010, no complications or deaths occurred. Of the fatalities, two had prostate cancer and one had multiple myeloma. All had tumors around 20 mm in size in the U/M with extended ESD times (up to 300 min). Two underwent non‐curative resections. The patient with perforation had surgery but died from disseminated intravascular coagulation (DIC) and uncontrolled bleeding. All three developed DIC and died within 30 days of ESD. Eleven patients died of primary advanced cancer within 4 years. The oldest patient was 90 years old and underwent ESD. Among patients who died of the original malignant tumor, five of 11 patients underwent non‐curative ESD for EGC, but none died from EGC.

**TABLE 4:** Clinicopathological features and clinical outcomes of three patients who experienced ESD‐related death.

Case	Date (year)	Age (years)	PS	Advanced malignant tumor	Stage	Size (mm)	Location	Procedure time (min)	Major histology	Depth	Adverse event	Survival period (days)
1	2007	78	1	Prostate	IVb	10	U	120	Undifferentiated	SM2	Delayed bleeding	14
2	2008	79	0	Multiple myeloma	III	20	M	300	Undifferentiated	SM1	Delayed perforation	30
3	2010	87	1	Prostate	IVb	22	U	40	Differentiated	M	Postoperative pneumonia	14

Abbreviations: ESD, endoscopic submucosal dissection; M, middle; M, intramucosal; SM, submucosal invasive; U, upper.

Results of the comparative analysis of the clinicopathological characteristics between cases complicated by DIC and those without DIC are presented in Table 5. No significant differences were observed with respect to age, sex, lesion location, major history, tumor depth, lesion size, or procedure time; only the subgroup of patients who underwent ESD prior to 2010 exhibited a statistically significant difference.

**TABLE 5:** DIC group versus non‐DIC group.

	DIC group, *n* = 3	Non DIC group, *n* = 23	*p*
Median age, years [range]	79.0 [78–87]	75.0 [50–90]	N.S
Sex, male	3 (100)	18 (78)	N.S
ESD performed before 2010	3 (100)	2 (9)	< 0.05
Location			0.123
Upper	2 (67)	4 (18)	
Middle & lower	1 (33)	19 (82)	
Major history			0.319
Differentiated typed	2 (67)	21 (91)	
Undifferentiated typed	1 (33)	2 (7)	
Depth of tumor (pathological T1a)	1 (33)	21 (91)	0.085
Median size, mm [range]	20 [5–110]	20 [10–22]	N.S
Median, procedure time, min [range]	72 [10–300]	120 [40–300]	N.S

Abbreviations: DIC, disseminated intravascular coagulation; ESD, endoscopic submucosal dissection.

Clinicopathological features and outcomes of non‐curative resections are presented in Table 6. Among 10 non‐curative ESD cases, two patients survived for > 5 years. None of these patients exhibited lymphovascular invasion or recurrence of GC.

**TABLE 6:** Clinicopathological features and clinical outcomes of 10 patients that underwent non‐curative resection by ESD for early gastric cancer.

Case	Age (years)	PS	Sex	Advanced malignant tumor	Stage	Location	Size (mm)	Macroscopic type	Procedure time (min)	Major histology	Depth	Outcome	Survival period (days)
1	68	1	M	Esophagus	IVb	L	30	IIa	120	Differentiated	SM2	Original	188
2	50	0	M	Esophagus	II	M	35	IIc	300	Differentiated	M (VM1)	Survival	3211
3	75	1	M	Esophagus	IVb	L	25	IIa	30	Differentiated	SM2	Original	500
4	74	1	M	Renal	IV	L	40	IIc	110	Undifferentiated	M	Original	261
5	77	0	M	Prostate	IVb	M	15	IIc + IIa	80	Differentiated	SM2	Original	855
6	78	1	M	Prostate	IVb	U	10	IIa	120	Differentiated	SM2	ESD related	14
7	81	1	M	Prostate	IVb	U	30	I	30	Differentiated	SM2	Original	28
8	56	0	F	Colorectal	IVa	M	110	IIa + I	300	Differentiated	M (UL1)	Survival	1865
9	79	0	M	Multiple myeloma	III	M	20	IIc	300	Undifferentiated	SM1	ESD related	30
10	53	0	F	Breast	IV	L	10	IIc	40	Undifferentiated	SM1	Original	1384

*Note:* No cases were lymphovascular invasion.

Abbreviations: Depth M, intramucosal; ESD; Endoscopic submucosal dissection; F, female; L, lower; M, male; M, middle; SM, submucosal invasive; U, upper; UL, Ulcerative findings; VM, Vertical margin.

Clinicopathological features and clinical outcomes of 11 patients who died of the original malignant tumor are presented in Table 7. They all died within 4 years; 5 of 11 patients had non‐curative ESD, but none had a recurrence of GC during the observation period.

**TABLE 7:** Clinicopathological features and clinical outcomes of 11 patients who died of the original malignant tumor.

Case	Age (years)	PS	Sex	Advanced malignant tumor	Stage	Location	Size (mm)	Macroscopic type	Procedure time (min)	Major histology	Depth	Survival period (days)
1	90	1	M	Hepatocellular	II	L	10	IIc	25	Differentiated	M	94
2	84	0	M	Hepatocellular	II	L	30	IIa	100	Differentiated	M	1133
3	77	0	M	Prostate	IVb	M	15	IIc + IIa	80	Differentiated	SM2	855
4	81	1	M	Prostate	IVb	U	30	I	30	Differentiated	SM2	28
5	68	1	M	Esophagus	IVa	L	30	IIa	120	Differentiated	SM2	188
6	75	1	M	Esophagus	IVb	L	25	IIa	30	Differentiated	SM2	500
7	64	0	M	Lung	IVa	M	15	IIc	60	Differentiated	M	603
8	65	0	M	Lung	IVa	L	5	IIc	40	Differentiated	M	278
9	73	1	M	Lung	IVa	U	40	IIa	120	Differentiated	M	870
10	74	1	M	Renal	IV	L	40	IIc	110	Undifferentiated	M	261
11	53	0	F	Breast	IV	L	10	IIc	40	Undifferentiated	SM1	1384

Abbreviations: F, female; L, lower; M, intramucosal; M, male; M, middle; SM, submucosal invasive; U, upper.

## 4 Discussion

This study included 26 EGC patients with concurrent advanced malignant tumors of other organs, of 3703 GC patients who underwent ESD between February 2005 and June 2022. En bloc resection was achieved for all lesions; however, 10 lesions (38%) were classified as non‐curative. Despite this, no cases exhibited lymphovascular invasion or recurrent GC. Notably, three ESD‐related deaths occurred, all in cases performed before 2010.

The 5‐year survival and recurrence rates for appropriately treated EGC are generally reported as > 90% and 1.0%–11.2%, respectively [23]. Herein, curative resection was achieved in 16 of 26 patients. Among 10 patients with non‐curative resection, none experienced the recurrence of GC during a maximum follow‐up period of 3221 days. Thus, local resection by ESD may be significant for EGC patients with advanced malignancies in other organs.

In contrast, a study of 905 patients with no additional treatment after non‐curative resection by ESD for EGC reported a median survival of 28 months after ESD, with recurrence observed in 25 patients over a median follow‐up of 64 months [24]. Lymphovascular invasion has been identified as a risk factor for recurrence in several studies. For example, in a cohort of 159 patients with GC who did not achieve a cure after ESD, the median survival was 27.5 months, with underlying disease and lymphovascular invasion as independent prognostic factors [25]. Risk factors for GC recurrence include lymphovascular invasion, associated with a hazard ratio of 6.6 [26], and lymphatic invasion, with a hazard ratio of 5.23 [27]. Herein, the absence of lymphovascular invasion likely contributed to the lack of recurrence and death from GC.

Furthermore, prognosis data for non‐curative resections indicate that patients undergoing curative surgical resection have significantly better overall survival (OS) and disease‐specific survival (DSS) compared to those who do not receive additional treatment [27]. However, the 3‐year DSS difference between the groups was relatively small (99.4% vs. 98.7%) compared to the more pronounced difference in 3‐year OS (96.7% vs. 84.0%) [27]. Although predicting lymphovascular invasion before ESD remains challenging, our results suggest that non‐curative resections offer some benefit for local control in the absence of lymphovascular invasion. Careful consideration is required when deciding on additional surgery for non‐curative resections, considering surgical tolerance and pathological factors.

An important result of this study is that three of 26 patients (12%) experienced complications after ESD that led to DIC and subsequent death. Among these patients, one had stage III multiple myeloma, and two had stage IVb prostate cancer. Despite all patients having a performance status of PS 0–1, the complications resulted in fatalities. Although previous reports indicate that death from ESD complications is rare [7], the presence of advanced cancers in other organs may have heightened the risk of DIC in our cohort. Therefore, careful consideration is needed when performing ESD in patients with EGC complicated by other malignancies.

Adverse events observed herein included delayed bleeding, perforation, and postoperative pneumonia. Delayed bleeding occurred in a patient with chronic kidney disease following renal cell cancer resection. Although delayed perforation after ESD for EGC is infrequent, excessive electrocautery during hemostasis may contribute to its development. The duration of cautery for hemostasis is significantly longer in cases with perforation compared to cases without perforation [28]. In addition, the U is a significant factor associated with delayed perforation [28], which may be related to the higher bleeding rate owing to the larger diameter of the submucosal arteries in U to M. [29] Herein, the procedure time for delayed perforation lasted 300 min due to the challenging lesion location and severe bleeding requiring repeated hemostatic interventions. Although we lacked video data for assessing hemostatic time, frequent hemostatic measures may have contributed to the delayed perforation.

Postoperative pneumonia was also a concern. Multivariate analysis identified several independent risk factors: age ≥ 75 years (odds ratio [OR] 2.83), cerebrovascular disease (OR 3.60), COPD (OR 2.64), delirium (OR 5.32), and remnant stomach or gastric tube (OR 5.30) [11]. A retrospective study reported a 1.6% incidence of postoperative pneumonia [7], but a separate report [30] noted that 66.7% of patients with pneumonia on CT scans had no pneumonia on chest radiography, suggesting that some cases may be overlooked. Although the exact number is not known, a certain number of cases have occurred. Although postoperative pneumonia cannot be completely avoided, early detection is possible with proper investigation, and special attention should be paid to patients with these risk factors. Aspiration pneumonia is more likely if there is significant fluid reflux into the oral cavity during esophageal ESD [31]. Measures such as proper intraoperative oral suctioning and avoiding deep sedation are necessary to mitigate this risk. In our study, an 87‐year‐old patient with EGC in the cardiac region experienced postoperative pneumonia, possibly due to fluid reflux from an incision made at the gastroesophageal junction. The incision was made on the esophageal side, which may have resulted in fluid reflux into the oral cavity and postoperative pneumonia. Thus, it is important to emphasize that even in patients with good PS, ESD for EGC with advanced cancer of other organs and poor prognosis may be fatal if complications arise.

Curative resection was achieved in 16 out of 26 cases. Among the remaining 10 cases, local disease control was obtained in 9, except for one case with a positive deep margin, resulting in a favorable local control rate of 96.2% (25/26). Severe complications occurred in 3 cases treated before 2010. However, no severe complications were observed in the 23 cases treated after 2010. Advances in devices and techniques in recent years [32, 33, 34, 35, 36, 37] may have contributed to reducing the risk of complications. However, overall, severe life‐threatening complications were observed in 3 out of 26 cases (11.5%), indicating a relatively high incidence. Given that only three cases developed DIC, it remains challenging to draw definitive conclusions regarding the risk factors for severe complications from the present study findings. In one case, it is speculated that a prolonged procedure time may have contributed to the occurrence of delayed perforation. Therefore, in cases where a longer procedure time is anticipated based on the lesion location or tumor size, more careful consideration of the indication for ESD may be warranted. Additionally, although factors such as nutritional status, performance status, and comorbidities are presumed to be important, the small number of cases in this study precluded a clear determination of their causal relationships with the development of severe complications, such as DIC. Thus, further investigation with a larger cohort is necessary. As the natural history of untreated EGC shows a median progression time of 44 months, during which tumor growth may lead to bleeding or obstruction and thereby impair patient quality of life, we sought to evaluate the outcomes of ESD in a cohort whose concomitant advanced malignancies predict a 5‐year survival rate below 50%. While comparing these ESD‐treated patients to contemporaneous patients with similar clinical indications who declined ESD would provide valuable insight into the appropriateness of the procedure, our institution lacked a dedicated registry of non‐ESD cases.

We performed a retrospective chart review of patients with advanced cancer managed between 2012 and 2022. Within this group, we identified 17 individuals who met standard endoscopic criteria for ESD but did not undergo the procedure. In contrast to our ESD cohort, these non‐treated patients predominantly exhibited poor performance status (PS 0–1, 5 cases; PS 2–3, 12 cases) or were deemed to have an exceedingly poor prognosis owing to their advanced disease. Their mean survival was only 0.9 years. Although a small number of patients with preserved PS and stable comorbid malignancies declined ESD because of personal preference—and would have constituted an ideal comparison group—such cases were exceedingly rare. Given these circumstances, any direct comparison would be subject to profound selection bias. Consequently, we concluded that it was not feasible to include a non‐ESD control group for assessing the validity of ESD in early GC concomitant with advanced malignancy. Nevertheless, ESD safety has steadily improved in recent years. However, the presence of coexisting advanced malignancies may worsen outcomes in the event of complications. Therefore, careful patient selection and thorough informed consent are essential when considering this treatment.

This study has some limitations. First, this was a single‐center trial with a small sample size. Second, patients who developed complications were treated before 2010, and newer endoscopic techniques for preventing complications were not fully utilized. Third, data collection was challenging for patients who did not undergo ESD for EGC with advanced tumors, preventing a comparison of their prognosis with those who did undergo ESD.

In conclusion, due to the non‐negligible risk of DIC associated with ESD, it is imperative to thoroughly assess the patient's suitability for ESD treatment and obtain comprehensive informed consent before its implementation.

## Ethics Statement

This article does not contain any studies with human or animal subjects performed by any of the authors.

## Consent

An opt‐out informed consent protocol was used for this study. The consent procedure was reviewed and approved by the Medical Ethics Committee of Hiroshima University (January 20, 2023; approval number [E2022‐0237]).

## Conflicts of Interest

The authors declare no conflicts of interest.

## Data Availability

The data that support the findings of this study are available on request from the corresponding author. The data are not publicly available due to privacy or ethical restrictions.

## References

[1] I. Uyama , K. Suda , M. Nakauchi , et al., “Clinical Advantages of Robotic Gastrectomy for Clinical Stage I/II Gastric Cancer: A Multi‐Institutional Prospective Single‐Arm Study,” Gastric Cancer 22 (2019): 377–385. 30506394 10.1007/s10120-018-00906-8

[2] J. Brahmer , K. L. Reckamp , P. Baas , et al., “Nivolumab Versus Docetaxel in Advanced Squamous‐Cell Non‐Small‐Cell Lung Cancer,” New England Journal of Medicine 373 (2015): 123–135. 26028407 10.1056/NEJMoa1504627 PMC4681400

[3] M. Davis , D. Hui , A. Davies , et al., “MASCC Antiemetics in Advanced Cancer Updated Guideline,” Support Care Cancer 29 (2021): 8097–8107. 34398289 10.1007/s00520-021-06437-w

[4] P. Rawla and A. Barsouk , “Epidemiology of Gastric Cancer: Global Trends, Risk Factors and Prevention,” Przeglad Gastroenterologiczny 14 (2019): 26–38. 30944675 10.5114/pg.2018.80001 PMC6444111

[5] L. A. Torre , F. Bray , R. L. Siegel , J. Ferlay , J. Lortet‐Tieulent , and A. Jemal , “Global Cancer Statistics, 2012,” CA: A Cancer Journal for Clinicians 65 (2015): 87–108. 25651787 10.3322/caac.21262

[6] Y. Yoshifuku , S. Oka , S. Tanaka , et al., “Long‐Term Prognosis After Endoscopic Submucosal Dissection for Early Gastric Cancer in Super‐Elderly Patients,” Surgical Endoscopy 30 (2016): 4321–4329. 26850026 10.1007/s00464-016-4751-y

[7] T. Akasaka , T. Nishida , S. Tsutsui , et al., “Short‐Term Outcomes of Endoscopic Submucosal Dissection (ESD) for Early Gastric Neoplasm: Multicenter Survey by Osaka University ESD Study Group,” Digestive Endoscopy 23 (2011): 73–77. 21198921 10.1111/j.1443-1661.2010.01062.x

[8] S. Oh , J. Lee , H. Lee , et al., “Natural History of Gastric Cancer: Observational Study of Gastric Cancer Patients Not Treated During Follow‐Up,” Annals of Surgical Oncology 26 (2019): 1–7. 10.1245/s10434-019-07455-z 31190210

[9] H. Tsukuma , A. Oshima , H. Narahara , and T. Morii , “Natural History of Early Gastric Cancer: A Non‐Concurrent, Long Term, Follow Up Study,” Gut 47 (2000): 618–621. 11034575 10.1136/gut.47.5.618 PMC1728114

[10] S. H. Jeong , M. I. Park , H. H. Kim , S. J. Park , and W. Moon , “The Natural Course of Early Gastric Cancer,” Korean Journal of Gastroenterology 60 (2012): 224–228. 10.4166/kjg.2012.60.4.224 23089908

[11] M. Kato , T. Michida , A. Kusakabe , et al., “Safety and Short‐Term Outcomes of Endoscopic Submucosal Dissection for Early Gastric Cancer in Elderly Patients,” Endoscopy International Open 4 (2016): E521–E526. 27227108 10.1055/s-0042-102650 PMC4874799

[12] S. Warren and O. Gates , “Multiple Primary Malignant Tumors. A Survey of the Literature and Statistical Study,” American Journal of Cancer 16 (1932): 1358–1414.

[13] Cancer Registry and Statistics. Survival Cancer Information Service, National Cancer Center, Japan, accessed January 31 2024, https://ganjoho.jp/en/professional/statistics.

[14] Japanese Society for the Colon and Rectum , “Japanese Classification of Colorectal, Appendiceal, and Anal Carcinoma: The 3rd English Edition [Secondary Publication],” Journal of Anus, Rectum and Colon 3 (2019): 175–195. 10.23922/jarc.2019-018 PMC6845287 31768468

[15] M. Kudo , Y. Kawamura , K. Hasegawa , et al., “Management of Hepatocellular Carcinoma in Japan: JSH Consensus Statements and Recommendations 2021 Update,” Liver Cancer 10 (2021): 181–223. 34239808 10.1159/000514174 PMC8237791

[16] J. L. Mohler , E. S. Antonarakis , A. J. Armstrong , et al., “Prostate Cancer. Version 2.2019, NCCN Clinical Practice Guidelines in Oncology,” Journal of the National Comprehensive Cancer Network 17 (2019): 479–505. 31085757 10.6004/jnccn.2019.0023

[17] Japan Esophageal Society , “Japanese Classification of Esophageal Cancer, Tenth Edition: Part I,” Esophagus 6 (2009): 1–25. 10.1007/s10388-016-0551-7 PMC5222932 28111535

[18] J. D. Brierley , M. K. Gospodarowicz , and C. Wittekind , TNM Classification of Malignant Tumors, 8th ed. (Wiley‐Blackwell, 2016).

[19] A. Palumbo , H. A. Avet‐Loiseau , S. Oliva , et al., “Revised International Staging System for Multiple Myeloma: A Report From International Myeloma Working Group,” Journal of Clinical Oncology 33 (2015): 2863–2869. 26240224 10.1200/JCO.2015.61.2267 PMC4846284

[20] W. Munakata , T. Terauchi , D. Maruyama , and H. Nagai , “Revised Staging System for Malignant Lymphoma Based on the Lugano Classification,” Japanese Journal of Clinical Oncology 49 (2019): 895–900. 31504700 10.1093/jjco/hyz111

[21] Japanese Gastric Cancer Association , “Japanese Classification of Gastric Carcinoma. 3rd English Edition,” Gastric Cancer 14 (2011): 101–112. 21573743 10.1007/s10120-011-0041-5

[22] Japanese Gastric Cancer Association , “Japanese Gastric Cancer Treatment Guidelines 2021. 6th Edition,” Gastric Cancer 26 (2023): 1–25. 36342574 10.1007/s10120-022-01331-8 PMC9813208

[23] T. Namieno , K. Koito , T. Higashi , T. Shimamura , K. Yamashita , and Y. Kondo , “Tumor Recurrence Following Resection for Early Gastric Carcinoma and Its Implications for a Policy of Limited Resection,” World Journal of Surgery 22 (1998): 869–873. 9673561 10.1007/s002689900484

[24] K. Takizawa , W. Hatta , T. Gotoda , et al., “Recurrence Patterns and Outcomes of Salvage Surgery in Cases of Non‐Curative Endoscopic Submucosal Dissection Without Additional Radical Surgery for Early Gastric Cancer,” Digestion 99 (2019): 52–58. 30554228 10.1159/000494413

[25] J. Y. Ahn , H. Y. Jung , J. Y. Choi , et al., “Natural Course of Noncurative Endoscopic Resection of Differentiated Early Gastric Cancer,” Endoscopy 44 (2012): 1114–1120. 23188661 10.1055/s-0032-1325676

[26] H. Suzuki , I. Oda , S. Abe , et al., “Clinical Outcomes of Early Gastric Cancer Patients After Noncurative Endoscopic Submucosal Dissection in a Large Consecutive Patient Series,” Gastric Cancer 20 (2017): 679–689. 27722825 10.1007/s10120-016-0651-z

[27] W. Hatta , T. Gotoda , T. Oyama , et al., “Is Radical Surgery Necessary in all Patients Who Do Not Meet the Curative Criteria for Endoscopic Submucosal Dissection in Early Gastric Cancer? A Multi‐Center Retrospective Study in Japan,” Journal of Gastroenterology 52 (2017): 175–184. 27098174 10.1007/s00535-016-1210-4

[28] Y. Yamamoto , H. Nishisaki , H. Sakai , et al., “Clinical Factors of Delayed Perforation After Endoscopic Submucosal Dissection for Gastric Neoplasms,” Gastroenterology Research and Practice 2017 (2017): 7404613. 28894466 10.1155/2017/7404613 PMC5574302

[29] T. Toyokawa , T. Inaba , S. Omote , et al., “Risk Factors for Perforation and Delayed Bleeding Associated With Endoscopic Submucosal Dissection for Early Gastric Neoplasms: Analysis of 1123 Lesions,” Journal of Gastroenterology and Hepatology 27 (2012): 907–912. 22142449 10.1111/j.1440-1746.2011.07039.x

[30] J. Watari , T. Tomita , F. Toyoshima , et al., “The Incidence of “Silent” Free Air and Aspiration Pneumonia Detected by CT After Gastric Endoscopic Submucosal Dissection,” Gastrointestinal Endoscopy 76 (2012): 1116–1123. 23164512 10.1016/j.gie.2012.07.043

[31] W. Hatta , T. Koike , H. Okata , et al., “Continuous Liquid‐Suction Catheter Attachment for Endoscope Reduces Volume of Liquid Reflux to the Mouth in Esophageal Endoscopic Submucosal Dissection,” Digestive Endoscopy 31 (2019): 527–534. 30861606 10.1111/den.13392

[32] N. Kawata , H. Ono , K. Takizawa , et al., “Efficacy of Polyglycolic Acid Sheets and Fibrin Glue for Prevention of Bleeding After Gastric Endoscopic Submucosal Dissection in Patients Under Continued Antithrombotic Agents,” Gastric Cancer 21 (2018): 696–702. 29357012 10.1007/s10120-018-0791-4

[33] Y. Tsuji , M. Fujishiro , S. Kodashima , et al., “Polyglycolic Acid Sheets and Fibrin Glue Decrease the Risk of Bleeding After Endoscopic Submucosal Dissection of Gastric Neoplasms (With Video),” Gastrointestinal Endoscopy 81 (2015): 906–912. 25440679 10.1016/j.gie.2014.08.028

[34] K. D. Choi , H.‐Y. Jung , G. H. Lee , et al., “Application of Metal Hemoclips for Closure of Endoscopic Mucosal Resection‐Induced Ulcers of the Stomach to Prevent Delayed Bleeding,” Surgical Endoscopy 22 (2008): 1882–1886. 18270775 10.1007/s00464-008-9743-0

[35] B.‐I. Lee , B.‐W. Kim , H.‐K. Kim , et al., “Routine Mucosal Closure With a Detachable Snare and Clips After Endoscopic Submucosal Dissection for Gastric Epithelial Neoplasms: A Randomized Controlled Trial,” Gut Liver 5 (2011): 454–459. 22195243 10.5009/gnl.2011.5.4.454 PMC3240788

[36] S. V. Kantsevoy , M. Bitner , A. A. Mitrakov , and P. J. Thuluvath , “Endoscopic Suturing Closure of Large Mucosal Defects After Endoscopic Submucosal Dissection Is Technically Feasible, Fast, and Eliminates the Need for Hospitalization (With Videos),” Gastrointestinal Endoscopy 79 (2014): 503–507. 24332082 10.1016/j.gie.2013.10.051

[37] O. Goto , M. Sasaki , T. Akimoto , et al., “Endoscopic Hand‐Suturing for Defect Closure After Gastric Endoscopic Submucosal Dissection: A Pilot Study in Animals and in Humans,” Endoscopy 49 (2017): 792–797. 28561197 10.1055/s-0043-110668

